# Mechanical Properties of Full-Scale Wooden Beams Strengthened with Carbon-Fibre-Reinforced Polymer Sheets

**DOI:** 10.3390/ma17194917

**Published:** 2024-10-08

**Authors:** Michał Marcin Bakalarz

**Affiliations:** Department of Theory of Structures and Building Information Modeling (BIM), Faculty of Civil Engineering and Architecture, Kielce University of Technology, Al. Tysiaclecia Panstwa Polskiego 7, 25-314 Kielce, Poland; mbakalarz@tu.kielce.pl

**Keywords:** beam, bending, composite, carbon fibres, epoxy resin, fabrics, FRP, numerical analysis, reinforcement, wood

## Abstract

The strengthening, rehabilitation and repair of wooden beams and beams made of wood-based materials are still important scientific and technical issues. This is reflected, among other things, in the number of scientific articles appearing and the involvement of research centres around the world. This is also related to society’s growing belief in the importance of ecological and sustainable development. This article presents an overview of the latest work in this field and the results of our own research on strengthening solid wooden beams with carbon-fibre-reinforced polymer (CFRP) sheets. The tests were carried out on full-size solid beams with nominal dimensions of 70 × 170 × 3300 mm. A 0.333 mm thick CFRP sheet was used for reinforcement. The research analysed various reinforcement configurations and different reinforcement ratios. For the most effective solution, a 46% increase in load capacity, 35% stiffness and 249% ductility were achieved with a reinforcement ratio of 1.7%. Generally, the higher the reinforcement ratio and coverage of the surface of the wood, the higher the strengthening effectiveness. The brittle fracture of wood in the tensile zone for unreinforced beams and the ductile crushing of wood in the compressive zone for reinforced beams were obtained. The most important achievement of this work is the description of the static work of beams in previously unanalysed configurations of strengthening and the confirmation of their effectiveness. The described solutions should extend the life of existing wooden buildings and structures and increase the competitiveness of wooden-based structures. The results indicate that, from the point of view of optimizing the cost of reinforcement, it is crucial to develop cheaper ways of combining wood and composite than to verify different types of fibres.

## 1. Introduction

Wood is one of the oldest construction materials known to humanity. The material is both ecological and environmentally friendly and has a beneficial effect on users. However, it is characterised by a wide variety of mechanical properties, anisotropic structure, the occurrence of defects, limitations in dimensions and susceptibility to the influence of external factors [1]. Consequently, due to these factors, they are susceptible to impairment or damage. Furthermore, the extension of the lifespan of building structures could create a necessity to increase their load-bearing capacity. These instances serve as the impetus for exploring novel repair, rehabilitation or reinforcement techniques [2].

In the existing timber structures, the augmentation of their load-bearing capacity is achievable by incorporating supplementary elements into the structure [3]. This cooperation relies on mechanical, adhesive or mechanical-adhesive connections [4]. Traditionally, these components manifested as sections and bars made of steel or aluminium [5]. Presently, composite materials are being employed owing to their numerous advantages. Compared to steel, composites’ main advantages include reduced weight, the ease of shaping and machining, elevated mechanical properties and good corrosion resistance. Predominantly utilised composites are those reinforced with aramid [6], basalt [7], glass [8] and carbon fibres [9]. Reinforcement is applied through various forms such as bars [10], sections (flat bars, T-sections, L-sections, etc.), technical fabrics [11], sheets, cords or meshes. These materials offer numerous advantages, such as increased strength, stiffness and flexibility, which can reduce the cost and weight of structures, the size of cross-sections or the use of cheaper, faster-growing wood species. Composite materials, on the other hand, have a high price per unit. Therefore, some authors resort to optimisation treatments to reduce the total cost of reinforcement. This can be achieved by using less-expensive glass fibres, combining different types of fibres (hybrids) [11] or reinforcing only the part subject to the most significant stress. The issue of sustainable construction, which can be achieved through mechanical joints, is also raised in some papers. In this way, it is possible to recover the components.

Borri and Corradi investigated the possibility of reinforcing wooden beams with high-strength steel meshes [12]. Compared to the reference beams, they achieved a significant load-bearing capacity and stiffness gains. They found that reinforcing can bind local defects in the form of crack, knot or gain discontinuities. Kossakowski and Sokolowski [13] used FRCM-PBO (fibre-reinforced cementitious matrix–p-phenylene benzobis oxazole) meshes to reinforce small wooden beams. The results showed a significant increase in the load-bearing capacity and ductility. At the same time, the stiffness of the composite section was not significantly affected. The reinforcement has also been designed to protect against fire. Nowak et al. [14] confirmed the validity of using CFRP laminates bonded inside the cross-section. They presented this procedure as a solution for elements of historical importance, where preserving the external surface in its original condition was necessary. Although a considerable scatter exists in the test results, an improvement in the mechanical properties of the reinforced beams was obtained. That is due to the natural character of wood. This author and Jerzy Jasieńko extended these analyses in their following paper [15], using steel flat plates as reinforcement. This increased load-bearing capacity is comparable to that achieved with the FRP (fibre-reinforced polymer) material. However, a significant increase in bending stiffness was achieved. Balmori et al. suggest reinforcing duo-glued timber beams with glass-fibre-reinforced polymer (GFRP) composite [16]. Although the material is less expensive, the increments in the stiffness and load-bearing capacity of reinforced beams are also lower compared to when carbon fibre would be used. The effect of the length of CFRP laminates on the load-bearing capacity and stiffness of reinforced beams was validated in the work referenced in [17]. It was observed that there was an increase in these parameters, although there was no significant correlation between the length of the reinforcement and the increase in stiffness. It is possible to combine several types of sheets within a single element to enhance the flexibility of reinforced beams, as demonstrated in [18]. Furthermore, this allows for a reduction in the costs of reinforcement. The number of reinforcement layers has been shown to influence the flexural resistance of the section, as illustrated in [19]. Nevertheless, the study’s authors identified that an increase in the number of layers could potentially lead to the reinforcement peeling off—provided that the epoxy resin should be selected carefully. It is also essential to consider that the reinforced elements are still susceptible to environmental stress. The study [20] concluded that ecological exposure can significantly reduce the load-bearing capacity, stiffness or ductility of reinforced elements.

In addition to the beams, several authors have also attempted to reinforce the timber columns. Abdallah et al. [21] examined the potential of utilising sprayed glass reinforcement. The variables under investigation in this study were the length and thickness of the reinforcement squirt. High effectiveness was attained. The durability of components reinforced with composites is discussed in detail in [22]. In addition to other tests, the authors conducted compressive strength tests on full-scale-reinforced elements. The most prevalent form of destruction observed was buckling, which can be attributed to the slenderness of the tested components. Thaeri et al. [23] presented a study on the feasibility of strengthening glue-laminated timber columns with FRP sheets. A partial covering of wood was advised from the cost point of view. A full covering was suggested for relatively deep cross-sections. Brol and Nowak [24] presented the results of compression tests of elements with varying slenderness and different types of damages strengthened with CFRP tapes and matting. The authors do not recommend complete wrapping, as it prevents a visual inspection of the wood. The strengthening effectiveness depended on the degree and type of material used.

In conclusion, incorporating composite reinforcement positively impacts the static behaviour of timber beams. The influence of the composite in question relies upon several parameters, including its mechanical properties (tensile strength, the modulus of elasticity and elongation at destruction), how it is manufactured (form), the degree of the reinforcement of the cross-section, its position about the cross-section and the method of joining.

The numerous advantages of timber, coupled with an increasing sense of environmental awareness, should increase the use of this material in the construction industry. Moreover, it should also contribute to prolonging the lifespan of the existing wooden structures. Consequently, it is imperative to implement measures that enhance their adaptability. The main objective of this study is to develop a composite wood CFRP section that can compete in the area of its physical and mechanical properties with other structural materials, such as reinforced concrete or steel. The simplicity of the reinforcement configurations presented allows for their utilisation in the reinforcement of the existing timber structures. This will also facilitate the retention of existing wooden buildings and structures in service for extended periods [25,26]. The present study encompasses a variety of reinforcement configurations. However, most importantly, the paper’s final section presents an analysis of the costs required to carry out each of them.

This paper analyses the static performance of unreinforced and reinforced flexural solid beams to achieve the stated objectives. The reinforcement of external surfaces was achieved using CFRP sheets bonded with epoxy resin adhesive. The variables subject to analysis were the degree of the reinforcement of the cross-section and the method of covering the external surfaces. The reinforcement was applied manually. The studies were conducted within the framework of the so-called four-point flexural testing method.

The selection of the reinforcing material was informed by the author’s previous research in reinforcing laminated veneer lumber (LVL) beams with carbon laminates and sheets, as discussed in the works [27,28,29]. The sheets offer several advantages over laminates glued inside grooved channels or to the external surfaces. One of the advantages is higher reinforcing effectiveness in terms of load-bearing capacity, as well as stiffness and ductility increases—when a similar reinforcement ratio is applied. Secondly, the exceptional quality of the composite wood bond is a notable feature—it significantly reduces the likelihood of failure due to delamination or debonding from the wood. The typical failure LVL beams reinforced with near-surface-mounted (NSM) CFRP laminate involved the destruction of adhesive and surrounding wood [28]. When CFRP laminate was bonded to the external face of the LVL beam, the failure was caused by the debonding [27]. In contrast to the CFRP-sheet-reinforced beams, the failure was due to the wood failure or rupture of composite material [29]. No debonding occurred—the comparison of selected models for predicting intermediate crack debonding failure was presented in [30] and the reviewed phenomenon of debonding was described in [31,32]. Furthermore, the sheet is flexible and readily adapted to uneven reinforced surfaces, making it easy to treat. Concurrently, it is a commonly available product in numerous developed countries.

## 2. Materials and Methods

### 2.1. Materials

#### 2.1.1. Wood

The subjects of the experimental research were fir beams with nominal dimensions of 70 × 170 × 3300 mm, purchased from Scanwood Scandinavian Wood Ltd. (Mikołów, Poland) [33]. A total of 25 beams were subjected to testing and divided into five-element series to ensure that the average volumetric density in each was approximately equal (Table 1). The wood’s volumetric density was determined before the reinforcement’s application. The reason behind such division was the desire to obtain uniform distribution of results, as wood elastic properties depend on its density [34]. The moisture content of the wood was estimated following the testing procedure outlined in the WRD-100 electrofusion moisture meter from TANEL Elektronika i Informatyka Spółka Jawna (Gliwice, Poland) [35], with the average of three measurements taken for each element (Table 1). This resulted in a relatively uniform distribution between series. The beams were stored in the laboratory hall before undergoing testing.

For beams lacking any form of reinforcement (series A), the flexural strength *f_m_* and global modulus of elasticity *E_m_*_,*g*_ were determined by the provisions outlined in EN 408 + A1:2012 [36]. The mean flexural strength was 48.12 MPa, with a standard deviation of 8.93 MPa. The mean elastic modulus was 11.56 GPa, with a standard deviation of 1.67 GPa. The strength class of the timber, as defined by EN 338:2016-06 [37], was C24—the primary determining factor was the bending stiffness of the wood. The flexural strength value is significantly greater than the value for this class. The results of the bending tests will be presented in greater detail in Section 3 of this article.

#### 2.1.2. Carbon-Fibre-Reinforced Polymer Sheets

The beams were reinforced with unidirectional carbon fibre CFRP sheets purchased from S&P Reinforcement Polska (Malbork, Poland) [38]. The sheets were delivered in 30 cm wide rolls and were subsequently cut to the requisite dimensions using scissors. The thickness of one and two layers of sheet was 0.333 and 0.666 mm, respectively. The principal axis of the sheet was parallel to the longitudinal axis of the beams. Table 2 presents the selected physical and mechanical properties of the composite.

**Figure 1:**
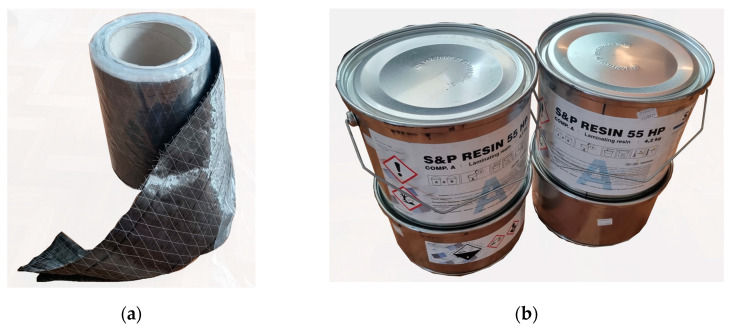
The reinforcement components comprise (**a**) a roll of carbon-fibre-reinforced polymer (CFRP) sheet and (**b**) an adhesive (author’s photograph).

Figure 1 presents a schematic representation of the reinforcement system kit. The carbon-fibre-reinforced polymer (CFRP) sheet comprises black carbon fibres oriented in the longitudinal direction, stabilised by white polyester fibres oriented in the transverse direction. The material is flexible, allowing it to conform to the surface of the component to be reinforced. Furthermore, it is straightforward to work with.

#### 2.1.3. Adhesive

The S&P Resin 55 HP two-component epoxy resin adhesive, purchased from S&P Reinforcement Polska (Malbork, Poland) [40], was employed to bond the sheets. The adhesive was supplied in 6 kg tins. Subsequently, the adhesive was mixed using a slow-speed mixer for approximately five minutes and then applied to the sheet’s surface (pre-impregnation) and the timber beam. The adhesive consumption of the prepared reinforcement area was approximately 1 kg per 1 square meter of sheet. Rubber spatulas were used to apply the adhesive and the sheet’s surface. The distribution direction was consistent with the main direction of the sheet. Table 3 presents the selected physical and mechanical properties of the adhesive.

### 2.2. Methods

Experimental studies were conducted at the Kielce University of Technology, employing the PN-EN 408 [36] standard as a reference point. The static scheme of the so-called four-point flexure test was implemented. The bending process was regulated by the movement speed of the loading thrust, which was assumed to be 7 mm/min. The preload in each actuator was approximately 0.2 kN. The tests were conducted until either failure or a significant reduction in the loading force was observed. 

**Figure 2:**
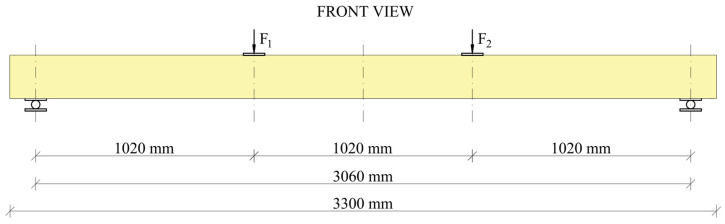
Test bench diagram.

Figure 2 shows the test bench diagram. The distance between the axis of the concentrated force and the axis of the nearest support was 1020 mm, which is six times the height of the cross-section. The distance between the points of concentrated force was also 1020 mm. The span at the support axes was 3060 mm. The overall length of the beam was 3300 millimetres.

**Figure 3:**
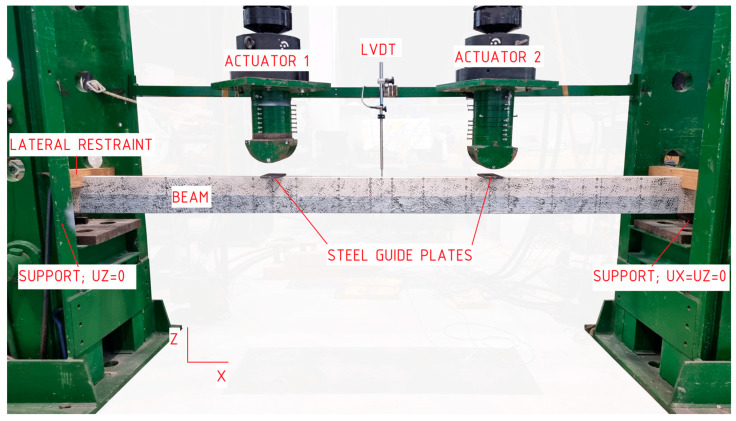
The following photograph, taken by the author, provides an overview of the test site.

Figure 3 shows the view of the test stand. The test stand comprised two actuators from Instron (Opole, Poland) independently controlled using RS BasTest software [41], steel guide plates positioned on the supports and at the point of application of concentrated forces, wooden lateral supports constructed from suitably shaped scantlings placed on the supports and a linear displacement sensor LVDT attached to an angle bracket at the back of the stand (part of HBM—Hottinger Baldwin Messtechnik system [42]). Hinged support on the right side and roller support on the left side were provided.

**Figure 4:**
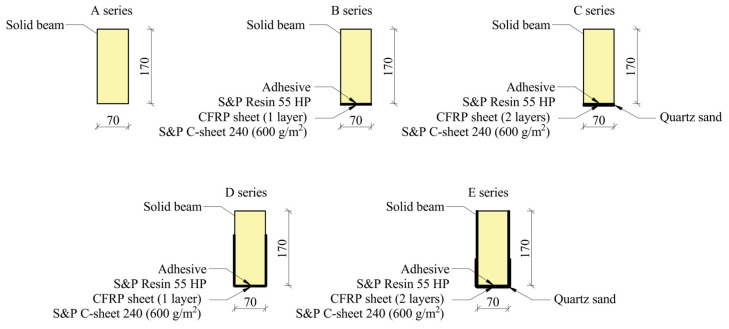
The following photograph, taken by the author, provides an overview of the test site.

The study encompassed the preparation of five research series (Figure 4). A total of five unreinforced beams and twenty reinforced beams were subject to testing. The individual series configurations are designated as follows:

- A—unreinforced beams;
- B—the beams were reinforced with a single layer of a 7 cm wide sheet bonded to the bottom surface (reinforcement ratio pt = 0.196%);
- C—beams reinforced with two layers of sheet, each measuring 7 cm in width, bonded to the lower surface (pt = 0.392%);
- D—beams reinforced with a single layer of 30 cm wide sheet bonded to the bottom and side surfaces (pt = 0.839%);
- E—beams reinforced with two layers of sheet, each 30 cm wide, bonded to the bottom (pt = 1.203%) and side surfaces, with overlap in the tension zone (pc = 0.476%).

The thickness of one and two layers of reinforcement was 0.333 and 0.666 mm, respectively.

During the tests, the loading force *F*, the test duration *t* and the actuator displacement were continuously measured using a computerised system. At the same time, the deflection at mid-span was monitored using an inductive sensor *u*. Following the bending test, photographic documentation was created, and the failure mode was described in detail. The measurements were recorded at a frequency of 5 Hz.

## 3. Results and Discussion

The beams’ bending behaviour is described in terms of load-bearing capacity, stiffness, ductility in bending, and the mode of failure. Furthermore, the potential for utilising a simple numerical model to anticipate the elastic behaviour of beams is explored, and the economics of the reinforcement methods are presented.

### 3.1. Load-Bearing Capacity

**Figure 5:**
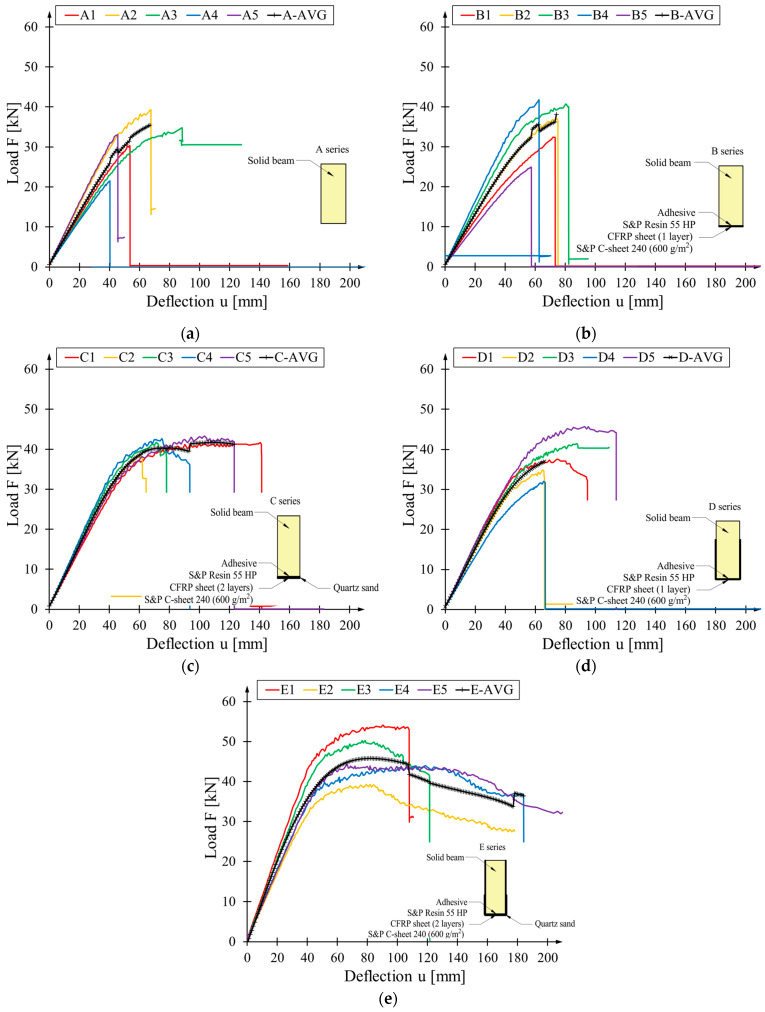
The load-bending relationship graph is presented below: (**a**) series A; (**b**) series B; (**c**) series C; (**d**) series D; (**e**) series E.

The load-deflection diagrams for the beams tested are shown in Figure 5. The behaviour from the start of the test to failure is almost linear for weakly reinforced and unreinforced beams (series A and B). The most significant variation in the slope of the curves in the elastic range is also characteristic of these series. These differences were caused by the wood nature. Wood is a natural material with non-uniformly distributed flaws along its volume. Furthermore, wood properties are dependent on many factors like density [34], which are different between elements in each series (Table 1). An increase in the reinforcement level accompanies the appearance of the plastic part of the graph. This is most evident In the E series for which CFRP sheets cover all the side surfaces. At the same time, the C series—two layers of sheet bonded to the underside—achieved the most stable bending test behaviour. 

The sharp drop in strength on the graphs is caused by the cracking of the wood, the rupture of the sheet or its detachment. These drops were not recorded for E2 and E5 beams. Their failure was due to the slow propagation of wood failure in the compression zone—wood crushing. The test was concluded when the maximum measurable deflection value at the centre of the beam span was achieved. In the case of beam D3, the appearance of the horizontal line indicates the beam’s destruction—the beam twisted, and so the sensor rested on the upper part of the beam did too. A comparable pattern of behaviour was observed in the case of the A3 beam.

Table 4, Table 5, Table 6, Table 7 and Table 8 show the detailed test results for the series tested. The values of the maximum loading force and the corresponding deflection value are shown. Their means and standard deviations are shown in the last columns. The type of failure mode, explained in Section 3.4, is shown in the bottom rows. The analysis of the curvatures in the diagrams leads to the conclusion that the value of deflection at maximum force is not a reliable parameter for describing the behaviour of flexural beams. The location of the peak of loading was not equal to the moment of the failure of the beam in most cases. In any case, the average deflection value for each reinforcement configuration is greater than that of the unreinforced beam.

**Figure 6:**
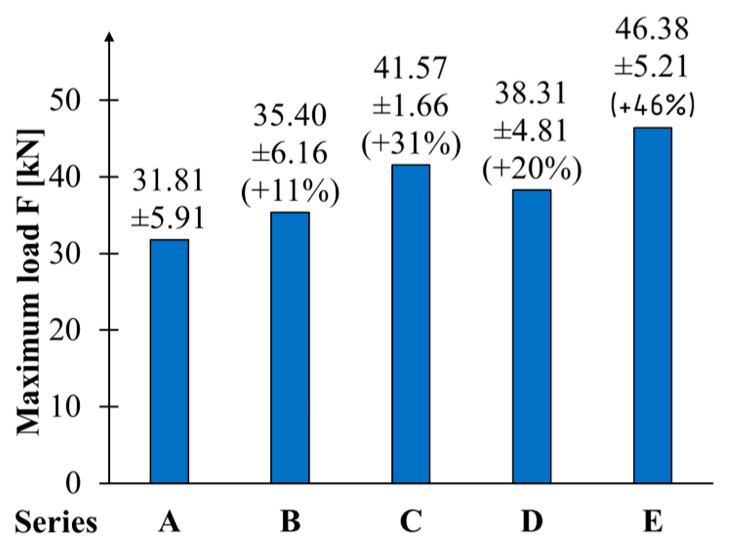
The mean values of the maximum load force.

Means and standard deviations for maximum force and relative increments for the series of reinforced beams are shown in Figure 6. The average failure force for the non-reinforced beams was 31.81 kN. The E-series beams, with a failure force of 46.38 kN, achieved the most significant increase in the capacity of 46%. Beams reinforced with a single layer of CFRP sheet (B series) showed the smallest growth in the load-bearing capacity of 11%. In this case, the maximum loading force was 35.40 kN. A significantly greater effectiveness of reinforcement was achieved by using it only on the underside (C series) compared to using twice as much but distributed over the bottom and sides (D series). Regarding the standard deviation of the breaking force, only the C-series beams showed a significant decrease with a deviation of 1.66 kN. The range fluctuated around 5–6 kN in the others.

The increments obtained are lower than those shown in the work of [43] for glued beams of small dimensions. The authors received an increment of approximately 50% of the flexural resistance using two layers of CFRP sheets. Work [29] demonstrated comparable improvements in load-bearing capacity when full-size beams of veneer glued in layers were reinforced with carbon-fibre-reinforced polymer (CFRP) sheets. The increase in load-bearing capacity was more than 50% for the E-type configuration. Additionally, applying CFRP sheets to the entire side surface was more effective. Liu et al. [44] obtained up to 110% increase in ultimate load for poplar beams strengthened with BFPR in comparison to control beams. 

Contrary to [45], doubling the reinforcing layers resulted in significantly increasing the effectiveness—almost three time when compared to one layer configuration. Similarly, the positive effect of doubling the number of reinforcing layers on bending behaviour was achieved by Zhang et al. [46]. The authors used hybrid-fibre-reinforced polymer (HFRP) sheets to strengthen rectangular and square timber beams. The bending ultimate bearing capacity increased by 20.2–36.4% for one layer and 41.9–72.6% for two layers of so-called optimized FRP in comparison to the unreinforced specimens.

### 3.2. Bending Stiffness

**Figure 7:**
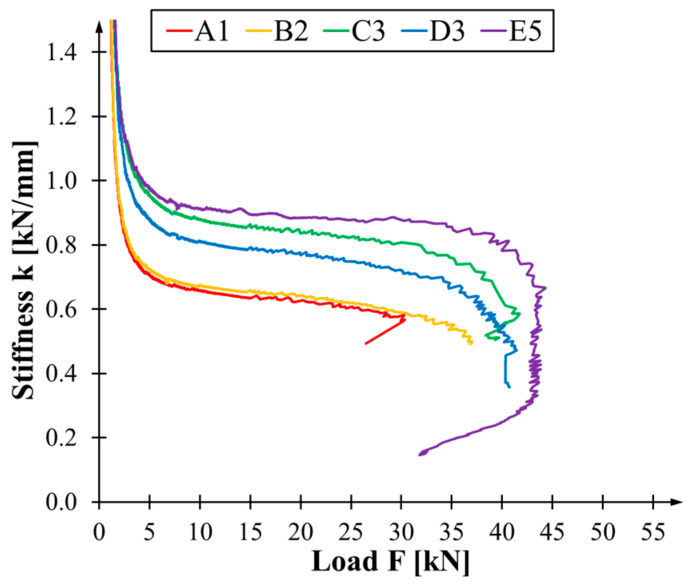
Bending stiffness versus load curves for selected specimens.

The bending stiffness coefficient *k* was estimated as the quotient of the loading force *F* and the deflection *u* by the formula [47]:

k = F/u,(1)

where F is the loading force in kilonewtons, u—the deflection at the centre of the beam span—is expressed in millimetres. Figure 7 plots the stiffness variation over time for a sample of specimens from each series. Stiffness decreases as load increases. The most rapid changes occur when starting and finishing bending tests. This is related to the beam stabilising or failing itself on the bench. For C3, D3 and E5 beams, the curves’ near-vertical or vertical slope is typical behaviour when the beam fails in the compression zone—either by crushing or buckling. This phenomenon corresponds to the plastic part of the load-deflection relationship in the diagram (Figure 5). This is not visible for A1 and B2 beams, and the curve ends at the point of fracture in the tensile zone, be it wood or wood and composite. Generally, the diagram of reinforced beams with an apparent plastic phase is similar in shape to a cotangent. For beams lacking any form of reinforcement, the curve can be described as a reciprocal function for positive values of the variable.

**Figure 8:**
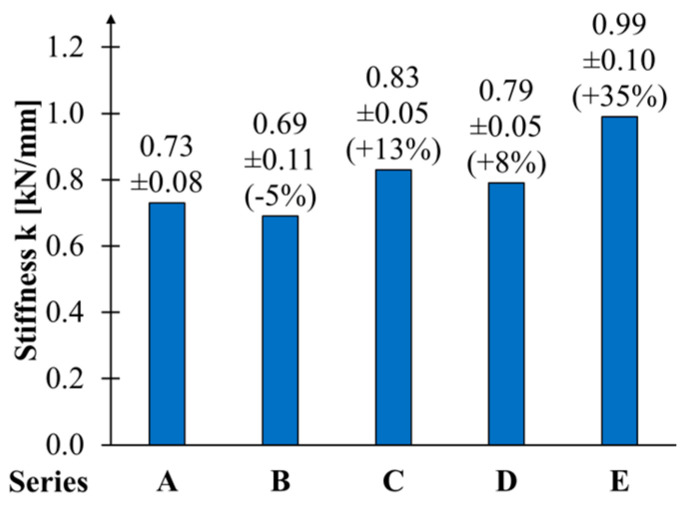
The average stiffness coefficient values.

A comparison of the average bending stiffness values of reinforced beams with those of unreinforced beams is presented in Figure 8. In light of the phenomenon above, of stiffness variation, the average value was calculated for a load range of 0.1 to 0.4 of the maximum force. This range is also employed in standards [36] to assess the bending modulus of the elasticity of timber beams. The typical behaviour involved increased bending stiffness with a growing ratio of cross-section reinforcement. The average k-factor values for the A, B, C, D and E series were 0.73, 0.69, 0.83, 0.79 and 0.99, respectively. This resulted in an average change in stiffness values of −5% to +35%.

The results demonstrate that only the last reinforcement configuration permitted a notable increase in stiffness during the initial phase of the flexural beams. In the case of beams reinforced with a single sheet layer, a decrease in stiffness was observed, although this result seems to be controversial. This phenomenon is attributable to the anisotropic structure of wood and the stochastic distribution of defects found in wood.

This also highlights the issue of the inadequacy of the comparison of the reinforced elements. Thus, it appears prudent to ascertain the beams’ original stiffness before applying reinforcement. This can be achieved by loading and unloading the beams within the elastic range. In this instance, the component may sustain initial damage due to an incorrectly defined load range.

Similar stiffness increments were obtained by Andor et al. [48] for the Norway spruce beams strengthened with two layers of CFRP fabric—equal to approx. 16%. For one layer, the value was approx. 15%—significantly higher than presented here. The effect of reinforcement ration on flexural stiffness was unclear. An increase in stiffness between 11% and 21% for glue-laminated timber beams strengthened with basalt and glass slack was mentioned by Thorhallsson et al. in the paper [49]. The pre-stressing of reinforcement does not affect stiffness significantly.

### 3.3. Ductility

Ductility is defined as a component’s capacity to withstand plastic deformation without reducing its load-bearing capacity before failure. This quantity can be determined by applying coefficients based on deformation comparisons or energy parameters. Given the shape of the load-bending equilibrium paths, a factor based on absorbed energy should be used according to the formula [47]:

µE = 1/2(Wtot/Wel + 1),(2)

where µE—ductility index, Wtot—total energy and Wel—elastic energy. The total and elastic energy values were calculated as the area under the load-bending relationship graph from the beginning of the test to the ultimate or proportionality limits. The mean values of these parameters are presented in Table 9.

The highest ductility coefficient µ_E_ of 5.94 was achieved for the E-series beams. The increment relative to the reference beams is 249%. The lowest for series B is 2.31. This represents a 6% reduction in value compared to the average for the A-series. A comparable value for the ductility coefficient was determined by Nadir et al. [43] for small, glue-laminated timber beams reinforced with one or two layers of composite sheets. Nevertheless, the authors observed a markedly lower ductility value for unreinforced elements. Higher increases (for U-type reinforcement), equal to 29.16% for the LVL beam strengthened with a CFRP sheet in comparison to series D, was presented by Subhani et al. [50].

### 3.4. Failure Mode

**Figure 9:**
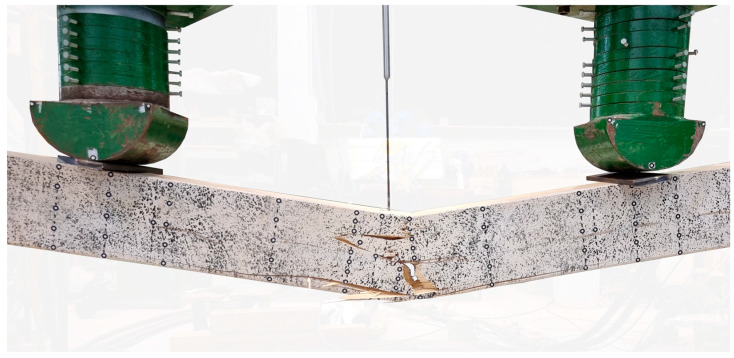
A typical example of a failure of an unreinforced element (photograph by the author).

The failure of the unreinforced elements was attributed to a brittle fracture initiated in the tensile zone at the centre of the cross-sectional span (Figure 9). It occurred immediately without signalling. Fracture propagated towards the upper surface of the beam. As a result, a reduction in effective cross-section high and load-bearing capacity was achieved. The fracture pattern differs significantly between beams and was influenced by the presence of wood flaws. No indentation of steel guide plates at supports and loading points occurred. This behaviour is typical for unreinforced elements in an air-dry state. 

The weakest point for unreinforced beams was tensile strength parallel to the grain of wood. Reinforcing this weakness resulted in more ductile and uniform bending behaviour. These changes, however, were dependent on the location and quantity of reinforcement. Reinforcement placed only on the underside did not bond the wood (and its flaws) on the sides and, therefore, no plasticization of the wood in tensile zone occurred. Covering the entire side surfaces of the wood with FRP resulted in a crushing of the wood in compressive zones—near to the loading axes. CFRP sheet bonded to external faces in the compressive zone is prone to buckling and debonding and, therefore, the utilization of its high mechanical properties is limited. Generally, from the quantity of reinforcement point of view, the higher the reinforcement ratio was, the more ductile the behaviour and the greater utilization of the compressive strength of wood that occurred. The used reinforcement ratio in the paper was not sufficient to prevent CFRP failure due to its rupture.

**Figure 10:**
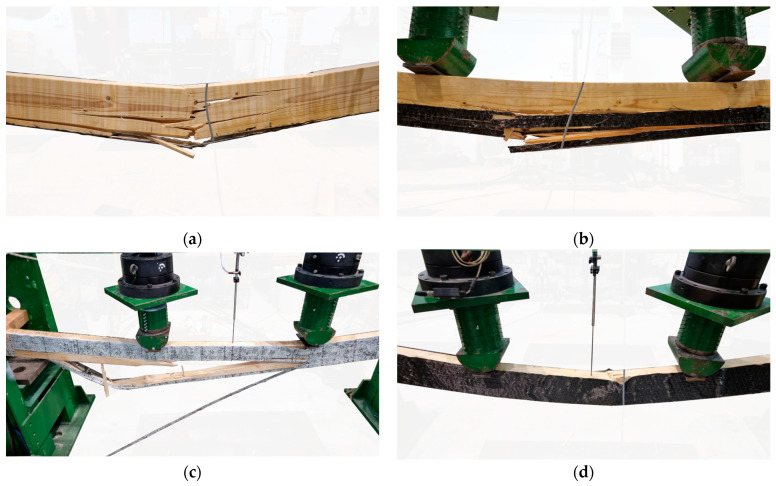
Failure mode of reinforced beams (photographs by the author): (**a**) timber cracking and sheet tearing; (**b**) timber cracking and detachment of the CFRP sheet; (**c**) timber cracking, sheet tearing in the tension zone and timber crushing in the compression zone; (**d**) timber crushing in the compression zone and buckling of reinforcement.

In the case of beams reinforced with one or two layers of sheet (B and C series) glued to the underside, the beam tended to fail due to the cracking of the timber and rupture of sheet (Figure 10a,b). In one instance, the sheet attached to beam C2 detached from the wood. Similar to [51], although the reinforcement improved the mechanical properties of the beams, the specimens tended to fail due to the flaws and low-tensile properties of the wood.

The typical failure mode of heavily reinforced elements (D and E-series beams) was the slow propagation of the plastic softening of the compression zone, which was usually accompanied by the breakage of reinforcement and the cracking of timber in the tension zone (Figure 10c). It was observed that wood crushing in the compression zone was frequently accompanied by the buckling of the CFRP sheet (Figure 10d). Only in the case of beams E2 and E5, the carbon composite was not ruptured. The crushing of the wood was accompanied by the “cracking” sounds.

In most of the cases analysed, the composite reinforcement was found to have been destroyed. Nevertheless, using a conservative degree of reinforcement in the tension zone of the cross-section, with a maximum of 1.203%, allowed for adding another layer to prevent this phenomenon from occurring. For LVL beams reinforced with sheets in similar configurations [29], a ratio of reinforcement of between 1.48 and 2.22% was found to provide sufficient protection.

Investigating the possibility of replacing the sheet in the compression zone with steel elements would be beneficial. The compressed sheets buckled (Figure 10d), rendering them of limited utility. Conversely, these materials increased the shear strength and the anchorage area.

### 3.5. Numerical Analysis

**Figure 11:**
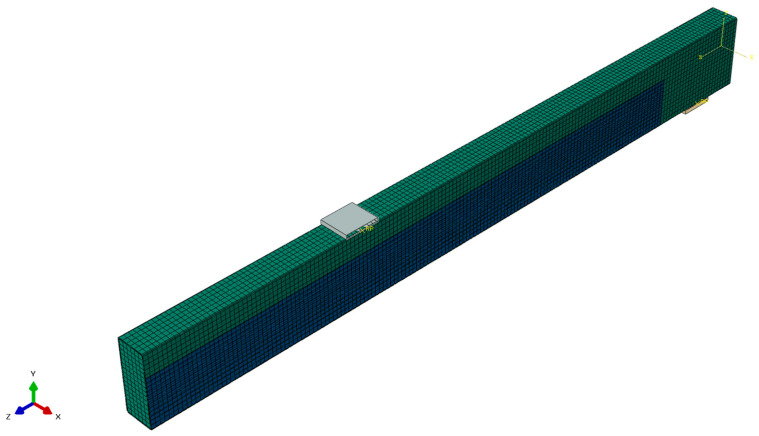
Meshed and assembled model of a B-series beam.

The numerical analysis was conducted utilising the standard module of the Abaqus 2017 software. The timber beams were modelled using 3D deformable element parts, the composite using 3D shell elements, and the supports as 3D analytical rigid elements. The data presented in Section 2.1.1 and Section 2.1.2 were used to create an elastic model of composite behaviour and an elastic-perfectly-plastic model for timber, respectively. The model was executed for half of the beam with a defined symmetry boundary condition assigned to the front surface—Z-SYMM (U3 = UR1 = UR2 = 0). The assembly comprised a single instance of a beam, a composite component and two supporting elements. The connection between the wood and the composite was established by utilising the tie function—negating the influence of the adhesive on the work. The contact properties between the elements and the supports were modelled using the “Hard Contact” function in the normal direction, with a friction coefficient of 0.3 in tangential direction. The interactions above were initiated via the “surface-to-surface contact” option. The beam’s loading was accomplished by applying a displacement at the reference point, which served as the loading. The calculations were executed within the elastic scope (Static, General). Figure 11 presents a view of the assembled and meshed model.

**Figure 12:**
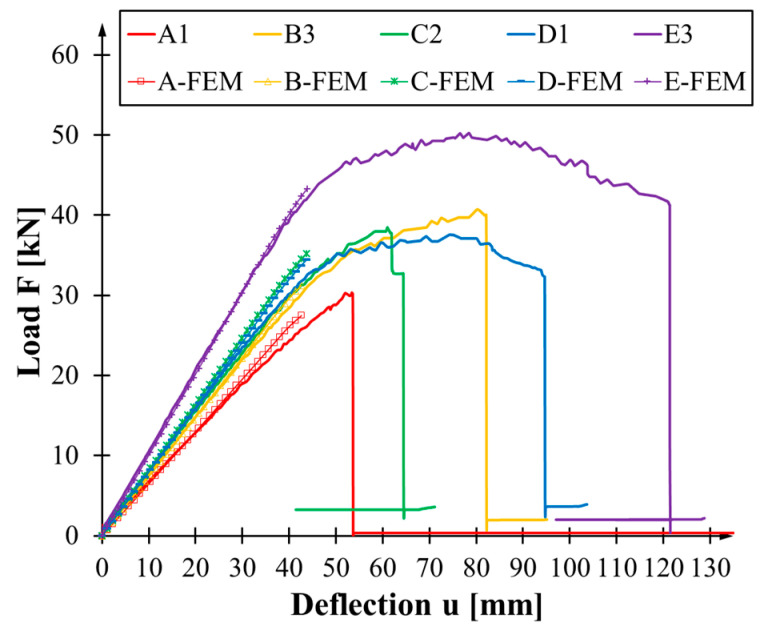
Comparison of experimental and numerical results.

The results of the numerical models are presented in Figure 12, which plots the curves obtained from the models against the experimental results for selected beams from each series. A high degree of concordance was observed between the experimental and numerical bending stiffness values. In the case of reinforced beams, the difference did not exceed 2%. In the case of unreinforced beams, the difference equalled 5%. Analogous outcomes have been observed in numerical simulations conducted under comparable assumptions. These include reinforcing beams made of laminated veneers that have been lumber reinforced with sheets [29] and laminates [27,28]. The transformed cross-section method also provides an approximate accuracy in estimating the bending stiffness. This method has been confirmed in numerous works, including [52,53].

The model, however, cannot predict the precise moment of destruction. This is attributable to the simplicity of the material models employed. The author plans to address the numerical modelling aspect of the failure of reinforced beams in a subsequent publication. Subsequent analyses will be enhanced by the incorporation of Hill’s plasticity criterion. As evidenced by numerous authors, such an extension permitted the accurate representation of the equilibrium paths of the unreinforced and reinforced elements. This is corroborated, among other sources, in [14,54,55]. It is now possible to create such models based on normative data or the literature. However, according to the author, it should result from his own research on wood and composites.

### 3.6. Economic Consideration

The following is an economic analysis of the implemented reinforcement configurations, considering the cost of purchasing the CFRP sheet and adhesive. However, the costs necessary for the preparation and application of reinforcement were omitted. The time required to apply the sheet and bind the adhesive was also not analysed. The material cost for reinforcement was approximately 128, 256, 548 and 1097 PLN net, respectively, for one beam of series B, C, D and E. In each subsequent series, the costs are twice as significant as the previous one. Only 23% of the total cost was for the CFRP sheet, while as much as 77% was for the adhesive. From an optimisation perspective, the critical element is developing a cheaper method for combining the composite and wood. Therefore, it may also be appropriate to consider using mechanical connectors instead of adhesive ones.

**Figure 13:**
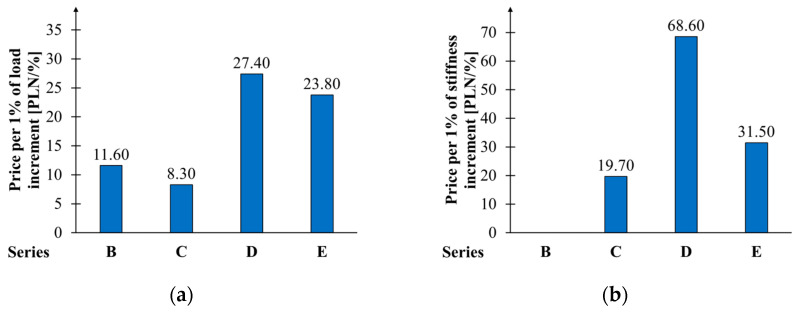
Economic reinforcement efficiency: (**a**) destructive force; (**b**) tensile strength.

Figure 13 shows the costs incurred to achieve a 1% relative increase in load capacity and bending stiffness. In the case of the B series, this parameter is not presented in the chart due to the lack of stiffness increase. The most economical solution is to apply two layers of CFRP sheet from the bottom (C series). Every percentage increase in load-bearing capacity costs 8 PLN, and the stiffness costs 19.6 PLN. The least economical solution is for the D series, for which these values are 27.4 and 68.6 PLN, respectively. For the most efficient configuration (E series), these costs are 23.8 and 31.5 PLN, respectively, for load capacity and stiffness.

## 4. Conclusions

This article presents the results of experimental and numerical studies on strengthening solid wooden beams using carbon sheets bonded to the external surfaces. Various beam reinforcement configurations were investigated. The reinforcement has positively affected the mechanical parameters of the beams. From an economic perspective, the most beneficial solution is to apply two sheet layers on the bottom face of the beam. In other cases, i.e., load capacity, stiffness and ductility, the most effective is the last configuration, where the CFRP sheet covers the entire side surface with an overlap in the tensile zone. With the increasing reinforcement ratio and coverage of the side surface, the effectiveness of reinforcement increases.

The article does not consider the influence of wood defects, long-term behaviour or the possibility of using local reinforcement at the location of damage. In the future, sheets will be used to reinforce locally occurring damage to wooden beams. It should be also noted that the presented study should be repeated in simulated real-world conditions under operational load as presented in [56].

The presented configurations may positively contribute to extending the lifespans of existing structures. They should allow for a change in the use of wooden structures where an increase in load capacity is necessary. 

To optimise the cost of reinforcement, the possibility of using alternative methods of connecting composite and wood should be verified. Given that the cost of sheets only constituted about 20% of the entire system, considering other (cheaper) fibres that would worsen reinforcement efficiency should not be considered. Mechanical fasteners as replaceable should be verified for that purpose.

## Figures and Tables

**Table 1:** The wood’s density and moisture content were determined through a series of tests.

Series	A (x ± s ^1^)	B (x ± s ^1^)	C (x ± s ^1^)	D (x ± s ^1^)	E (x ± s ^1^)
Density (kg/m^3^)	479.5 ± 23.5	479.3 ± 40.0	478.7 ± 33.3	479.0 ± 35.4	479.2 ± 49.2
Moisture content *w* (%)	13.7 ± 0.58	14.0 ± 0.36	14.1 ± 0.17	14.8 ± 0.47	13.5 ± 0.51

^1^ x—arithmetic mean; s—standard deviation.

**Table 2:** The physical and mechanical properties of CFRP (prepared based on [29] according to the manufacturer’s data [39]).

Series	Value
Modulus of elasticity *E_f_* (GPa)	265
Tensile strength *f_t_*_,*f*_ (MPa)	5100
Density *ρ_f_* (kg/m^3^)	1800
Elongation at rupture *ε_f_* (%)	1.7–1.9
Design thickness *t_f_* (mm)	0.333—one layer; 0.666—two layers

**Table 3:** The select physical and mechanical properties of CFRP (prepared based on [29] according to the manufacturer’s data [40]).

Series	Value
Bending strength *f_m_* (MPa)	85.3 ^1^
Modulus of elasticity *E_k_* (Mpa)	3200
Density *ρ_k_* (kg/m^3^)	1200–1300 (1150 ^1^)
Compressive strength *f_c_*_,*k*_ (Mpa)	100

^1^ Based on own experimental tests.

**Table 4:** Series A test results.

Parameter	Beam					Mean Value (Std. Dev.)
	A1	A2	A3	A4	A5	
*F_max_* (kN)	30.35	39.31	34.79	21.53	33.08	31.81 (5.91)
*u_max_* (mm)	53.41	67.55	88.33	40.27	45.52	59.02 (17.30)
Failure mode	Tension	Tension	Tension	Tension	Tension	

**Table 5:** Series B test results.

Parameter	Beam					Mean Value (Std. Dev.)
	B1	B2	B3	B4	B5	
*F_max_* (kN)	32.45	37.06	40.72	41.80	24.95	35.40 (6.16)
*u_max_* (mm)	71.96	74.32	80.20	62.47	57.28	69.25 (8.27)
Failure mode	Tension + FRP rupture	Tension + FRP rupture	Tension + FRP rupture	Tension + FRP rupture	Tension + FRP rupture	

**Table 6:** Series C test results.

Parameter	Beam					Mean Value (Std. Dev.)
	C1	C2	C3	C4	C5	
*F_max_* (kN)	41.70	38.48	41.72	42.68	43.29	41.57 (1.66)
*u_max_* (mm)	140.40	61.02	71.14	74.93	103.30	90.16 (28.77)
Failure mode	Tension + FRP rupture	Tension + FRP debonding	Tension + FRP rupture	Tension + FRP rupture	Tension + FRP rupture	

**Table 7:** Series D test results.

Parameter	Beam					Mean Value (Std. Dev.)
	D1	D2	D3	D4	D5	
*F_max_* (kN)	37.57	34.86	41.46	32.01	45.63	38.31 (4.81)
*u_max_* (mm)	74.46	65.02	87.63	65.49	92.85	77.09 (11.37)
Failure mode	Tension + Compression + FRP rupture	Tension + Compression + FRP rupture	Tension + Compression + FRP rupture	Tension + FRP rupture	Tension + Compression + FRP rupture	

**Table 8:** Series E test results.

Parameter	Beam					Mean Value (Std. Dev.)
	E1	E2	E3	E4	E5	
*F_max_* (kN)	54.13	39.28	50.24	43.97	44.29	46.38 (5.21)
*u_max_* (mm)	90.37	81.67	78.49	118.70	66.56	87.16 (17.52)
Failure mode	Tension + Compression + FRP rupture	Compression + FRP buckling	Tension + Compression + FRP rupture/buckling/debonding	Tension + Compression + FRP rupture/buckling	Compression + FRP buckling	

**Table 9:** Ductility analysis results.

Parameter	Series				
	A	B	C	D	E
Wel (J)	210.03 ± 109.24	430.58 ± 154.52	459.75 ± 49.88	407.23 ± 79.60	540.31 ± 88.11
Wtot (J)	1143.42 ± 547.67	1485.47 ± 429.38	2937.38 ± 1141.32	1850.97 ± 1204.61	5673.35 ± 1154.84
µE (-)	2.49 ± 0.27	2.31 ± 0.39	3.60 ± 0.91	2.71 ± 1.12	5.94 ± 1.50

## Data Availability

All data are available in the paper.
